# Supplementary material for: The Small RNA Universe of Capitella teleta
Source: Front Mol Biosci. 2022 Feb 25;9:802814. doi: 10.3389/fmolb.2022.802814 (PMC8915122; doi:10.3389/fmolb.2022.802814)
Supplement: Supplementary file 1 [file DataSheet1.ZIP › Supplement/known/CAPTEscaffold_12_1217.pdf]

Provisional ID	:	CAPTEscaffold_12_1217
Score total	:	3396
Score for star read(s)	:	3.9
Score for read counts	:	3385.9
Score for mfe	:	1.6
Score for randfold	:	1.6
Score for cons. seed	:	3
Total read count	:	6653
Mature read count	:	3995
Loop read count	:	0
Star read count	:	2658

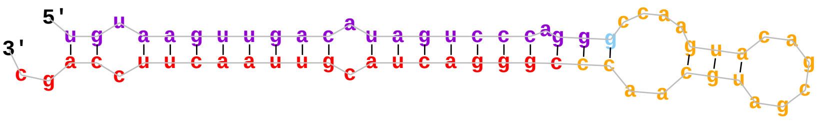

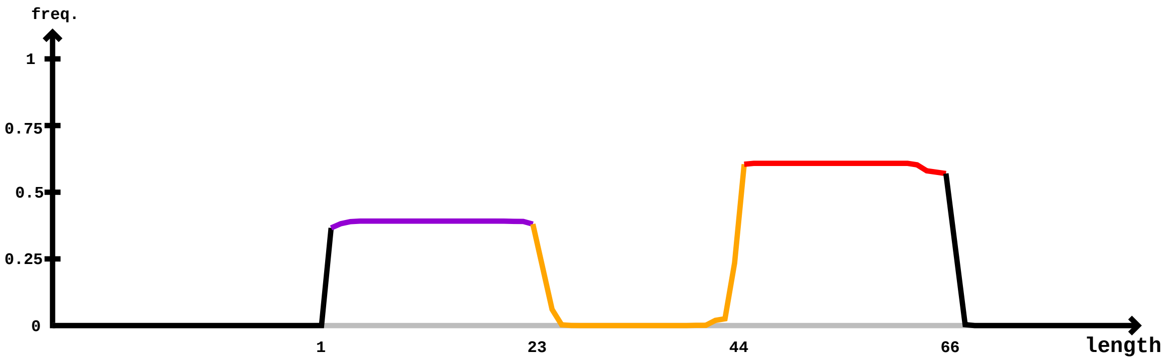


```
                                 Star                                        Mature
5'- auaaagaaggaaaaucgugagcgcaguuguaaguugacauagucccagggccaaguacagcgaugcaacccgggacuacguuaacuuccagccucgaacacggagcgacug  -3'   obs
    auaaagaaggaaaaucgugagcgcaguuguaaguugacauagucccagggccaaguacagcgaugcaacccgggacuacguuaacuuccagccucgaacacggagcgacug        exp
    auaaagaaggaaaaucgugagcgcaguuguaaguugacauagucccagggccaaguacagcgaugcaacccgggacuacguuaacuuccagccucgaacacggagcgacug        known
    .......((.....(((((..((..((((.((((((((.(((((((.(((....(((......)))..)))))))))).)))))))).))))..))..)))))).....)).    reads    mm    sample

    ..........................uuguaaguugacauagucccag...............................................................    1        0     seq
    ...........................uguaaguugacauagucc..................................................................    1        0     seq
    ...........................uguaaguugacauaguccc.................................................................    6        0     seq
    ...........................uguaaguugacauaguccca................................................................    2        0     seq
    ...........................uguaaguugacauagucccag...............................................................    65       0     seq
    ...........................Gguaaguugacauagucccag...............................................................    1        1     seq
    ...........................uguaaguugUcauagucccagg..............................................................    1        1     seq
    ...........................uguaaguugacauagucccagA..............................................................    2        1     seq
    ...........................uguaaguugacauagucccagU..............................................................    1        1     seq
    ...........................uguaaguugacauagucUcagg..............................................................    2        1     seq
    ...........................uguaaguugacauagucGcagg..............................................................    1        1     seq
    ...........................uguaaguugacauagAcccagg..............................................................    2        1     seq
    ...........................uguaaguugacauagucccagC..............................................................    1        1     seq
    ...........................Nguaaguugacauagucccagg..............................................................    1        1     seq
    ...........................uguaaguugacGuagucccagg..............................................................    1        1     seq
    ...........................uguaaguugacauaguccUagg..............................................................    1        1     seq
    ...........................uguaaguugacaAagucccagg..............................................................    1        1     seq
    ...........................uguaaguugacauagucccaAg..............................................................    1        1     seq
    ...........................uguaaguugacauagucccagg..............................................................    1671     0     seq
    ...........................Aguaaguugacauagucccagg..............................................................    9        1     seq
    ...........................Gguaaguugacauagucccagg..............................................................    2        1     seq
    ...........................ugAaaguugacauagucccagg..............................................................    5        1     seq
    ...........................uguaaguugacauaguGccagg..............................................................    1        1     seq
    ...........................uguGaguugacauagucccagg..............................................................    1        1     seq
    ...........................ugGaaguugacauagucccagg..............................................................    1        1     seq
    ...........................uguaaguugacauagucccaggA.............................................................    60       1     seq
    ...........................ugAaaguugacauagucccaggg.............................................................    3        1     seq
    ...........................uguaaguugacauagucccagAg.............................................................    1        1     seq
    ...........................Aguaaguugacauagucccaggg.............................................................    2        1     seq
    ...........................uguaaguugacauagucccaggC.............................................................    2        1     seq
    ...........................uguaaguugacauagucccaggg.............................................................    368      0     seq
```

```
                           Star                                         Mature
auaaagaaggaaaaucgugagcgcaguuguaaguugacauagucccagggccaaguacagcgaugcaacccgggacuacguuaacuuccagccucgaacacggagcgacug
...........................Nguaaguugacauagucccaggg.............................................................    1     1   seq
...........................uguaaguugacauagucccaggU.............................................................    9     1   seq
...........................uCuaaguugacauagucccagggc............................................................    1     1   seq
...........................uguaaguugacauagucccagggc............................................................    138   0   seq
...........................uguaaguugacauagucccagggU............................................................    39    1   seq
...........................uguaaguugacauagucccagggA............................................................    76    1   seq
...........................uguaaguugacauagucccagggcc...........................................................    1     0   seq
...........................uguaaguugacauagucccagggcU...........................................................    3     1   seq
............................guaaguugacauagucccagg..............................................................    3     0   seq
............................guaaguugacauagucccaggg.............................................................    12    0   seq
............................guaaguugacauagucccagggA............................................................    2     1   seq
............................guaaguugacauagucccagggU............................................................    2     1   seq
............................guaaguugacauagucccagggc............................................................    87    0   seq
.............................uaaguugacauagucccagg..............................................................    1     0   seq
.............................uaaguugacauagucccaggg.............................................................    4     0   seq
.............................uaaguugacauagucccagggU............................................................    7     1   seq
.............................uaaguugacGuagucccagggc............................................................    2     1   seq
.............................uaaguugacauagucccagggc............................................................    28    0   seq
.............................Aaaguugacauagucccagggcc...........................................................    1     1   seq
.............................uaaguugacauagucccagggcc...........................................................    9     0   seq
.............................uaaguugacauagucccagggcU...........................................................    1     1   seq
..............................aaguugacauagucccagggc............................................................    11    0   seq
..............................aaguugacauagucccagggcc...........................................................    1     0   seq
..............................aaguugacauagucccagggccU..........................................................    2     1   seq
.................................................................caacccgggacuacguuaacuuc.......................    3     0   seq
.................................................................caacccgggacuacguuaacuucc......................    3     0   seq
...................................................................Gcccgggacuacguuaacuuc.......................    1     1   seq
...................................................................aAccgggacuacguuaacuuc.......................    1     1   seq
...................................................................acccgggacuacguuaacuuc.......................    25    0   seq
...................................................................acccgggacAacguuaacuucc......................    1     1   seq
...................................................................Gcccgggacuacguuaacuucc......................    1     1   seq
...................................................................acccgggacuacguuaacuucc......................    89    0   seq
...................................................................acccgggacuacguuaacuuAc......................    1     1   seq
...................................................................acccgggacuacguuaacuucU......................    4     1   seq
...................................................................acccggCacuacguuaacuucc......................    1     1   seq
...................................................................acccgggacuacguuaacuuccU.....................    2     1   seq
....................................................................cccgggacuacguuaacuuc.......................    1     0   seq
....................................................................cccgggacuacguuaacuucc......................    38    0   seq
....................................................................cccgggacuacguuaacuucca.....................    2     0   seq
.....................................................................ccgggacuacguuaacuuc.......................    2     0   seq
.....................................................................ccgggacuacguuaacuucc......................    9     0   seq
.....................................................................ccgggacuacguuaacuucca.....................    26    0   seq
.....................................................................cGgggacuacguuaacuucca.....................    1     1   seq
.....................................................................ccgggacuacguuaacuuccag....................    29    0   seq
.....................................................................ccgggacuacguuaacuuccagc...................    1054  0   seq
.....................................................................ccgggacuacguuaacuucAagc...................    2     1   seq
.....................................................................ccgggaGuacguuaacuuccagc...................    1     1   seq
.....................................................................ccgggacuacguuaacuuccagA...................    27    1   seq
.....................................................................ccgggacuacguuaGcuuccagc...................    1     1   seq
.....................................................................ccgggacuacgAuaacuuccagc...................    1     1   seq
.....................................................................ccgggacuacguuaacAuccagc...................    1     1   seq
.....................................................................ccgggacAacguuaacuuccagc...................    1     1   seq
.....................................................................ccgggacuaAguuaacuuccagc...................    1     1   seq
.....................................................................Acgggacuacguuaacuuccagc...................    1     1   seq
.....................................................................ccgggacuacguuaacuuccagU...................    211   1   seq
.....................................................................ccgggacuacguuaaAuuccagc...................    1     1   seq
.....................................................................ccgggaUuacguuaacuuccagc...................    1     1   seq
.....................................................................ccgggacuacguuaacuuGcagc...................    1     1   seq
.....................................................................ccgggUcuacguuaacuuccagc...................    1     1   seq
.....................................................................ccgggacuacgGuaacuuccagc...................    1     1   seq
.....................................................................ccAggacuacguuaacuuccagc...................    3     1   seq
.....................................................................ccgAgacuacguuaacuuccagc...................    4     1   seq
.....................................................................ccgggacuacguuaacuuccagcA..................    3     1   seq
.....................................................................ccgggacuacguuaacuuccagcU..................    34    1   seq
.....................................................................ccgggacuacguuaacuuccagccu.................    1     0   seq
.....................................................................ccgggacuacguuaacuuccagcUu.................    1     1   seq
......................................................................cgggacuacguuaacuuc.......................    5     0   seq
......................................................................cgggacuacguuaacuucc......................    5     0   seq
......................................................................cgggacuacguuaacuucca.....................    3     0   seq
......................................................................cgggacuacguuaacuuccag....................    6     0   seq
```

```
                           Star                                         Mature
auaaagaaggaaaaucgugagcgcaguuguaaguugacauagucccagggccaaguacagcgaugcaacccgggacuacguuaacuuccagccucgaacacggagcgacug
......................................................................cgggacuacguuaacuuccagA...................    7    1    seq
......................................................................cgggacuacguuaacuuUcagc...................    1    1    seq
......................................................................cgggacuacguuaacCuccagc...................    1    1    seq
......................................................................cAggacuacguuaacuuccagc...................    6    1    seq
......................................................................Ugggacuacguuaacuuccagc...................    1    1    seq
......................................................................cggAacuacguuaacuuccagc...................    1    1    seq
......................................................................cgggacCacguuaacuuccagc...................    1    1    seq
......................................................................cgggacuacguuaacuuccagG...................    1    1    seq
......................................................................Ggggacuacguuaacuuccagc...................    1    1    seq
......................................................................cgggacuacguuaacuuccagU...................    4    1    seq
......................................................................cgggGcuacguuaacuuccagc...................    1    1    seq
......................................................................cgggacuacguuaacuucUagc...................    7    1    seq
......................................................................cgggacuacgAuaacuuccagc...................    1    1    seq
......................................................................cgggacuaUguuaacuuccagc...................    1    1    seq
......................................................................cgAgacuacguuaacuuccagc...................    6    1    seq
......................................................................cgggacuacguuaacuuccagc...................    2277 0    seq
......................................................................cgggacuacguuaacuuAcagc...................    1    1    seq
......................................................................cgggacuacguAaacuuccagc...................    2    1    seq
......................................................................cgggacuacguuaacuuccagcc..................    2    0    seq
......................................................................cgggacuacguuaacuuccagcU..................    162  1    seq
......................................................................cgggacuacguuaacuuccagcA..................    3    1    seq
......................................................................cgggacuacguuaacuuccagcUu.................    6    1    seq
.......................................................................gggacuacguuaacuuccagc...................    6    0    seq
.......................................................................gggGcuacguuaacuuccagc...................    1    1    seq
.......................................................................gggGcuacguuaacuuccagccu.................    1    1    seq
.......................................................................gggacuacguuaacuuccagccu.................    15   0    seq
```
